# Bone Status and Early Nutrition in Preterm Newborns with and without Intrauterine Growth Restriction

**DOI:** 10.3390/nu15224753

**Published:** 2023-11-11

**Authors:** Marta Meneghelli, Andrea Peruzzo, Elena Priante, Maria Elena Cavicchiolo, Luca Bonadies, Laura Moschino, Francesca De Terlizzi, Giovanna Verlato

**Affiliations:** 1 Neonatal Intensive Care Unit, Department of Women’s and Children’s Health, University Hospital of Padova, 35128 Padova, Italy; marta.meneghelli@aopd.veneto.it (M.M.); andrea.peruzzo.3@studenti.unipd.it (A.P.); elena.priante@aopd.veneto.it (E.P.); mariaelena.cavicchiolo@aopd.veneto.it (M.E.C.); luca.bonadies@aopd.veneto.it (L.B.); laura.moschino@aopd.veneto.it (L.M.); 2 Laboratory of Clinical Biophysics, IGEA, 41012 Modena, Italy; f.deterlizzi@igeamedical.com; 3 Paediatric Nutrition Service, Department of Women’s and Children’s Health, University Hospital of Padova, 35128 Padova, Italy

**Keywords:** preterm newborn, intrauterine growth restriction, bone status, nutrition

## Abstract

Intrauterine growth restriction (IUGR) together with preterm birth could be harmful to bone health. The aim of the study was to examine bone status in IUGR versus non-IUGR preterms and to analyze the nutritional management best correlated with its improvement. Newborns < 34 weeks of gestational age (wGA), 75 IUGR and 75 non-IUGR, admitted to the Neonatal Intensive Care Unit of the University Hospital of Padova were enrolled and monitored from birth until 36 wGA through anthropometry (weight, length, head circumference, lower limb length (LLL)), biochemistry, bone quantitative ultrasound assessment of bone status (metacarpus bone transmission time, mc-BTT, us) and nutritional intakes monitoring during parenteral nutrition. IUGR compared to non-IUGR showed lower mean mc-BTT (0.45 vs. 0.51, *p* = 0.0005) and plasmatic phosphate (1.45 vs. 1.79, *p* < 0.001) at birth. Mc-BTT at 36 wGA, though equal between groups, correlated in IUGR newborns with basal phosphate, mean total energy of the first week and month (positively) and days to reach full enteral feeding (negatively). Lower i.v. vitamin D intake, LLL and prolonged total parenteral nutrition predicted worse mc-BTT at 36 wGA in the enrolled infants. These results suggest that preterms and in particular IUGR newborns need special nutritional care to promote bone development.

## 1. Introduction

The goal of nutrition in preterm infants is to provide nutrients to enable their potential growth trajectory avoiding the possible deficiencies due to the premature interrupted placental supply. In fact, preterm infants are invariably deprived of the benefits of the last trimester of pregnancy (mineralization combined with fetal movements against the uterine wall), and above all, preterm infants with low birth weight (LBW) [1].

Growing evidence suggests that LBW is associated with subnormal peak bone mass (PBM) and adults born preterm with very low birth weight (VLBW) display inferior bone mineral density (BMD) [2].

As Balasuriya et al. observed in their study, the attenuated PBM in LBW infants is of concern, because PBM is an important determinant of fracture risk later in life [3] and, worldwide, >11% of deliveries are preterm with survival rate of infants with VLBW increased to almost 90%, especially in developed countries [4].

The combination of Intrauterine Growth Restriction (IUGR) and deprivation of the third trimester could be especially harmful for bone health.

IUGR is estimated to affect between 5% and 10% of all pregnancies worldwide [5] and it refers to a condition in which a fetus is unable to achieve its genetically determined potential size. It is a prenatal finding of growth restriction, confirmed by a documented low fetal growth rate and/or by the presence of specific causes, such as fetal infection, genetic abnormalities and impaired placental blood flow or toxicities.

This condition can strongly influence the newborn’s short- and long-term outcome as IUGR preterm newborns are exposed to a higher risk of complications than the non-IUGR premature infants of the same gestational age (GA), such as impaired physical growth, cognitive, and motor development [6]. It is known that IUGR do not reach their potential growth due to altered placental transfer, not only of oxygen but also of nutrients, and this may result in several metabolic alterations like hypoglycemia and hypocalcemia [7]. In particular the relationship between impaired fetal growth and glucose metabolism disorders has long been known, also if only recently literature distinguishes in a clear way IUGR from Small for Gestational Age (SGA) newborn (newborn with an estimated fetal weight or birth weight below the 10th percentile compared to what is expected for a given gestational age) [8].

The accelerated postnatal growth and related insulin resistance is another characteristic of IUGR newborns [9], related to metabolic disorders later in life [10]. Furthermore, IUGR newborns have low fat mass [11], benefit from lower amino acids placental transfer [12] and have lower Fat Free Mass [13].

An impaired skeletal muscle development in IUGR was also reported in animal studies and related to altered protein synthesis [14].

For all these reasons they could be even more sensitive to nutrients provision, though the ESPGHAN committee proposed the same nutritional management as appropriate for gestational age infants (AGA) due to the lack of data [15].

An impaired fetal growth can also predispose to certain major diseases later in life, including metabolic syndrome and chronic lung and kidney diseases [16]. In fact, nutritional imbalance and growth restriction during critical windows of the fetal and neonatal life can lead to long-term developmental deficits and morbidity in later life and also to impairment of the skeletal growth trajectory.

Thus, premature birth, especially in very-low-birthweight infants (VLBWIs), and restricted growth patterns could reduce bone accretion and deteriorate peak bone mass, resulting in an increased risk of future osteoporosis [17,18].

Few data exist on IUGR bone status. In mouse models the uteroplacental insufficiency in the IUGR condition predisposes to an endochondral ossification deficit at birth due to cellular changes within the growth plate, associated with reduced length, mineralization and strength of bone [19], but these data are not confirmed by human studies. Furthermore, many studies have explored small for gestational age (SGA) infants, rather than IUGR, as risk factor for reduced BMD and bone mineral content (BMC) in the neonatal period in VLBWIs [20].

Therefore, we aimed to examine bone status at birth in IUGR newborns compared to non-IUGR newborns and to analyze the postnatal nutritional management that best correlates with its improvement.

## 2. Materials and Methods

### 2.1. Population

Patients admitted to the Neonatal Intensive Care Unit (NICU) of the Women’s and Children’s Health Department of University-Hospital of Padova were prospectively enrolled in the study if they were <34 weeks of gestational age (GA), receiving total parenteral nutrition (TPN) within 48 h of life with informed consent obtained in the first 48 h for participation of a legally acceptable representative. IUGR newborns were matched with non-IUGR infants with the same GA, born in the same month and with the least difference in date of birth and days of GA. Exclusion criteria were a major congenital abnormality or a chromosomal abnormality, known or suspected congenital metabolic disease, congenital infections, neonatal death within 7 days of life and refusal of consent.

### 2.2. Collection of Data

Patients were monitored from birth through anthropometric, clinical and biochemical parameters. For each enrolled subject the following data were collected: demographic data such as GA, birth weight, length, head circumference, lower limb length, number of newborns small for GA, sex, pregnancy, type of delivery, antenatal steroids administration, Apgar score at 5 min and intrauterine growth restriction diagnosed by expert obstetricians based on prenatal ultrasonographic anthropometric and doppler parameters [21].

Furthermore, for each enrolled subject, growth parameters during the neonatal intensive care unit (NICU) stay were collected. Growth was assessed through anthropometric measures: daily weight was registered and total length, head circumference, lower limb length at birth, 21 days of life and at 36 weeks of gestational age (wGA). A balance with the sensibility of one gram (Incubator 8000 SCVR, Drager- (Draeger Italia SpA, Milano, Italy) or Tassinari Bilance (5.9 S.R.L. Care Weighting System, Bologna, Italy)) was used to weigh preterm newborns. The total length and head circumference were obtained through a meter with the sensibility of 0.1 cm. Lower limb length was obtained in the same leg where mc-BTT was measured through a neonatal knemometer (Digital-Taschen-Messschieber, Messwelt, Hagen, Germany). Limb length was measured with the infant lying in the supine position, with 90° flexion at the hip and knee. Three readings for each measure were collected and the mean of these readings was calculated.

Days to regain birth weight, minimum weight, maximum weight loss, and days to reach 1800 g were also evaluated. Biochemical data were collected, particularly serum calcium, phosphate and alkaline phosphatase at birth and at least weekly until 21 days of life or until the parenteral nutrition was discontinued. To assess bone status, we performed quantitative ultrasound (QUS) with DBM Sonic BONE PROFILER (IGEA, Modena, Italy) within 48 h from birth, at 21 days of life and at 36 weeks of GA, which allows the evaluation of bone density, elasticity and bone structure. We tested the second metacarpus bone transmission time (mc-BTT, µs) as previously reported [22].

At 36 wGA extrauterine growth restricted infant was defined as an infant with weight below the 10th percentile according to the Fenton Preterm Growth Chart [23].

Standardized intravenous parenteral nutrition (glucose, ammino acids, lipids, minerals, electrolytes, vitamins and trace elements) was provided within 24 h of life following our protocol and in particular: ammino acids supplementation started at birth with 1.5–2 g/kg per day, advanced to a maximum of 3.5 g/kg per day with Non Proteic Energy started from 45 till 80 kcal/kg per day. Calcium and phosphorus intakes were between 0.8 and 2 mmol/kg per day. Parenteral magnesium and vitamin D administration were 0.15–0.2 mmol/kg and 30–160 IU/kg per day, respectively. The volume and number of feeds were decided by the attending physician. Briefly minimal enteral feeding was started in the first day of life, with human milk or formula then feedings were advanced at a rate of 10–20 mL/kg per day. As feeding advanced, TPN was decreased and stopped when enteral feeding reached 100–120 mL/kg per day.

Nutritional intakes during parenteral nutrition were collected such as energy, protein, phosphorus, calcium and vitamin D intakes. Total energy, defined as the sum of energy provided by parenteral and enteral nutrition, was also calculated. Days of parenteral nutrition, time to full enteral feeding (FEF, defined as 150 mL/kg/day), and type of feeding after PN and at discharge were also recorded.

Clinical outcomes were recorded such as incidence of respiratory distress syndrome (RDS), bronchopulmonary dysplasia (BPD), hemodynamically significant patent ductus arteriosus (PDA), intraventricular haemorrhage (IVH grade III or IV using the Papile classification), sepsis (documented or suspected, deterioration of clinical condition with positive blood culture), necrotizing enterocolitis (NEC, defined as Bell stage II or III), retinopathy of the prematurity (ROP, need for laser therapy), cholestasis (defined when direct bilirubin > 1 mg/dL if the total serum bilirubin is ≤5 mg/dL or >20% [24]), days of hospitalization, and death.

### 2.3. Analysis of Data

Statistical analysis has been conducted by software NCSS^®^ version 9.0 (NCSS 9 Statistical Software (2013),NCSS, LLC. Kaysville, UT, USA). Continuous variables were tested for normal distribution by applying the Shapiro–Wilk test before applying all subsequent statistical tests. Data were expressed as mean ± s.d. Student’s t-model was used to compare the different variables between the two groups under study (IUGR vs. non-IUGR). QUS data were analyzed both cross-sectionally and longitudinally, using univariate models. Pearson’s correlation was used to analyze relationships between variables. The existence of statistically significant differences between the two groups has been assessed by use of the chi-square for categorical variables. Finally, the median value of some variables was selected as a cut-off to divide the population into two groups whose mc-BTT at 36 GA was compared using Student’s *t*-test. A multivariate analysis of variance (MANOVA) was carried out in enrolled newborns in predicting/detecting the differences in the bone status. Significance was set at *p* < 0.05.

The study was approved by the Hospital Ethics Committee (reference number 4374/AO/17) and informed consent was obtained from the parents.

## 3. Results

### 3.1. Demographic and Clinical Data

Recruitment started January 2018 and lasted until December 2020. All subjects were recruited in the NICU of Women’s and Children’s Health Department of University-Hospital of Padova. A total of 354 preterm infants were enrolled and among these 81 were diagnosed with IUGR. A total of 75 IUGR were matched with 75 non-IUGR infants with the same GA and they were followed up until discharge from our unit. The main clinical features and auxological parameters at birth of the two cohorts of subjects are presented in Table 1.

### 3.2. IUGR vs. Non IUGR: Clinical, Biochemical, Nutritional and Bone Status Comparisons

At birth IUGR infants showed lower birth weight, total length, head circumference and lower limb length than non-IUGR infants (Table 1). No differences were detected about sex, multiple pregnancies, mode of delivery, prenatal steroids, or Apgar at 5 min between the two groups. As expected IUGR showed more days of ventilation and CPAP while the other clinical outcomes were similar. Moreover, we observed that at 36 weeks of GA, 98.6% of IUGR newborns showed extrauterine growth restriction for weight (Table 1).

During the NICU stay, they showed lower maximum weight loss, but they maintained a lower weight and more days to reach 1800 g. At term corrected age their weight, head circumference, total length and lower limb length continued to be lower (Table 2).

Analysis of biochemical data are reported in Table 2. Of note, IUGR infants showed a lower plasmatic phosphate at birth and 21 days of life and a higher plasmatic calcium at birth. Alkaline phosphatase (ALP) was higher in the reference population at baseline, but plasma concentrations remained within normal physiological ranges in both groups. In contrast, at 21 days of age ALP levels were higher in the IUGR group, but without reaching statistical significance (Table 2).

IUGR infants at birth and at 21 days of life had a worse bone status (lower mc-BTT, metacarpal bone transmission time), while no differences were detected at 36 weeks of GA (Table 2).

Regarding nutritional intake, IUGR infants received prolonged PN and lower mean energy intake during the first week of life, while mean total energy intake in the first month was lower without reaching statistical significance (*p* = 0.0518); protein intakes were not different (Table 3); IUGR also received higher phosphorus intakes in the first 2 weeks and lower iv vitamin D and calcium intakes in the first week (Table 3). Considering enteral nutrition, they took a longer time to reach full enteral feeding of 150 mL/kg per day (Table 3); no differences were detected in the use of human milk.

### 3.3. Newborns’ Bone Status and Correlations with Anthropometric and Clinical Parameters

We found a positive correlation between mc-BTT at birth and 36 weeks GA with the corresponding time of weight and lower limb length in IUGR newborns; in non-IUGR newborn basal mc-BTT correlated with birth weight (Table 4).

The correlations between nutritional and biochemical parameters with bone status, for the IUGR and non-IUGR groups, are reported in Table 5. In the IUGR group basal serum phosphate levels were positively correlated with mc-BTT at 21 days and 36 weeks GA, while alkaline phosphatase (ALP) levels at 21 days were negatively correlated with mc-BTT at 21 days. In the non-IUGR group basal phosphate was not correlated with mc-BTT while ALP negatively correlated with both determinations (21 days and 36 wGA).

Vitamin D iv intakes in the first week positively correlated with mc-BTT at 36 wGA in both groups. In both groups days of TPN negatively correlated with mc-BTT at 36 wGA while number of days needed to reach full enteral feeding (FEF, 150 mL/kg/day) only in the IUGR group negatively correlated with QUS values on day 21 and at 36 weeks of GA. Mc-BTT at 36 wGA, only in the IUGR preterms positively correlated with basal phosphate, mean total energy of the first week and month and negatively with days to reach FEF.

### 3.4. Markers of Bone Status in the Preterm Population

Searching for parameters that could be useful markers of bone status and using as the threshold value for each parameter the median of the distribution, mc-BTT at 36 weeks of GA was worse in newborns with lower limb length at 21 days < 91 mm, a mean energy intake in first week < 77 Kcal/kg/day or in first month of life < 106 Kcal/kg/day, an intravenous vitamin D intake in first week < 37 IU/Kg/day or a prolonged TPN (days ≥ 16) (Table 6).

Then we put together all the significant variables affecting bone health at 36 wGA to investigate which ones contribute independently to the mc-BTT value at 36 wGA with a multivariate analysis. TPN days, intravenously vitamin D intake in the first week of life and lower limb length at 21 days of life predicted bone status at 36 weeks of GA with the following model: BTT 36 weeks GA = 0.4962 + 0.0569 × (TPN days) + 0.0401 × (VitD iv 1week) + 0.0421 × (lower limb length 21 day) (Table 7).

A lower intravenously vitamin D intake in the first week, a lower limb length and prolonged TPN are associated with worse mc-BTT at 36 weeks of GA.

## 4. Discussion

Preterm infants are at risk for impaired bone mineralization and length later in life, due to the loss of last trimester mineral accumulation and the inadequate nutritional intake in the early postnatal period [25]. Improvement in early nutritional intake decreases the cumulative nutritional deficit and, thereby, may prevent growth retardation [26] and could also improve skeletal development [27,28].

Given the same GA, anthropometric parameters of IUGR infants (including lower limb length) are lower at birth than non-IUGR and additionally they have a lower mc-BTT at birth and at 21 days of life.

IUGR infants regain birth weight more quickly than non-IUGR infants; they reach a lower minimum weight during hospitalization but they take a longer time to reach 1800 g of body weight and have more days of hospitalization. The weight and total length of IUGR were lower in all measurement intervals, as also reported in the literature [29] and almost all IUGR newborns maintained extrauterine growth restriction. Rapid weight recovery in preterm infants is associated with better outcomes in terms of growth and neurocognitive development, but also with an increased risk in later life of developing metabolic syndrome [30]. Despite this, Embleton et al. [31] demonstrated that metabolic disorders occur mainly when growth recovery takes place after one year of age, which is why current guidelines promote aggressive nutrition to favor rapid postnatal growth.

Analysis of biochemical data showed in IUGR infants a lower plasmatic phosphate at birth, which can be related to an insufficient prenatal transfer to the fetus [32] and it can represent a sign of malnutrition as it happens in the refeeding syndrome [33]. IUGR infants in fact are malnourished during intrauterine life: at birth, with the start of TPN, the insulin level increases and promotes the entry of phosphate in the cells, inducing hypophosphatemia and hypokaliemia in an anabolic phase in which requests are high [34]. At birth IUGR newborns had not only lower phosphorus but also higher calcium levels. A possible explanation could be that in case of hypophosphatemia, phosphorus is released from the bone and therefore calcium is also mobilized, leading to higher calcium levels [33]. These results outline once more the importance of ensuring to these newborns not only adequate amounts of calories but also of minerals to promote bone growth. Phosphorus is ubiquitous and plays a key role in maintaining cellular homeostasis, in the composition of phospholipids, nucleotides, nucleic acids [35] and also in bone development.

Of note, we found a statistically significant positive correlation between basal serum phosphate levels and mc-BTT at baseline and at 36 weeks of GA only in the group of IUGR newborns; additionally, phosphoremia at 21 days of life remains lower in IUGR and it is positively associated with BTT at 36 weeks of GA. These data suggest a role for phosphatemia in the first weeks of life in predicting in-patient bone status, with higher blood phosphorus levels being associated with better bone health, as reported in the literature [36].

Furthermore, if mc-BTT values at baseline and at three weeks of life are lower in IUGRs than in non-IUGRs, since mc-BTT is closely associated with bone health, it can be inferred that being born IUGR is a risk factor for worse bone status at birth and in the first weeks of life [37].

IUGR newborns received longer parenteral nutrition and reached full enteral feeding later. These data could reflect the caution of neonatologists in introducing enteral feeding due to the possible association between NEC and intrauterine growth restriction [38]. Nonetheless, the latest European guidelines recommend that enteral nutrition should be started as early as possible for the preterm infant, with the aim of reducing the duration of parenteral nutrition and limiting the time needed to achieve exclusive enteral nutrition [15]. Of note in our study the clinical outcomes, including NEC, were not different in the two groups apart from days of ventilation/CPAP that were increased, as expected, in the IUGR group. It is noteworthy that only in the IUGR group, days to reach FEF negatively correlated with mc-BTT, outlining the necessity of improving this nutritional practice especially in IUGR preterms.

Searching for nutritional variables that influenced bone status in IUGR infants, we found that prolonged periods of TPN correlate negatively with mc-BTT at three weeks of life and at 36 weeks of GA in both groups. In fact, prolonged periods of parenteral nutrition are associated with lower protein and energy intake, which may impair the synthesis of new bone tissue [39], while the poor mineral solubility in parenteral solutions could lead to inadequate intake and result in lower phosphatemia to support bone growth rates [22,23,24,25,26,27,28,29,30,31,32,33,34,35,36,37,38,39,40].

Indeed, IUGR newborns received lower energy intake on average in the first week and in the first month of life (though the latter without significance), which could contribute to worsen bone status, as a better bone condition regards infants fed with higher energy intake from the first days of life [39]. Interestingly, energy intake in the first week positively correlated with bone status specifically in the IUGR group.

A previous study conducted by our group [28] demonstrated that neonates fed with higher protein and energy intakes in the first weeks of life showed better bone status at 21 days of life and at 36 weeks of GA as assessed by mc-BTT. The nutritional intake of proteins, energy and minerals plays a significant role in the correct bone development and metabolism, as it contributes to the proper formation of the structural organic matrix and to the regulation of growth hormone and IGF-I, which are involved in the synthesis of bone in the prepubertal age [41]. It has been hypothesized that peak bone mass during child skeletal growth may be a predictor of osteoporosis at a later age. Furthermore, according to the hypothesis of the early origins of adult diseases, IUGR infants may also experience intrauterine conditions during fetal life that interfere with postnatal bone status programming. An early nutritional intervention aimed at preventing alterations in bone status in the newborn, already beginning in the pregnant mother and continuing in the baby in postnatal life, could result in better outcomes also in adults.

Higher intakes of vitamin D during the first week of life were associated with better ultrasonographic parameters at 36 wGA in both groups. Vitamin D is an important regulator of phospho-calcium metabolism. However, there is not a unique consensus in the literature on the vitamin D intake required for health bone condition [42,43]. Nowadays guidelines recommend a vitamin D intake of 80–400 IU/Kg/day by parenteral nutrition and between 400–700 IU/Kg/day up to a maximum of 1000 IU/day enterally, while in the past lower intakes were recommended [44]

Searching for parameters that could be useful markers of bone status in all the enrolled patients, as mc-BTT was equal in both groups, we found that mc-BTT at 36 weeks of GA was worse in newborns that received a vitamin D intake in first week < 37 UI/Kg/day. Also, preterm newborns with a mean energy intake in first week < 77 Kcal/kg/die or in first month of life < 106 Kcal/kg/die, or with a prolonged TPN (days ≥16) had a worse mc-BTT at 36 weeks of GA.

Interestingly, newborns with lower limb length at 21 days < 91 mm had a significantly worse mc-BTT at 36 weeks GA. Lower limb length was previously correlated with bone status at birth [45] and during hospitalization [27]. It would be interesting, in a larger group of patients, to verify if lower limb length could be used as marker of bone development.

It was finally possible to use a multivariate analysis of variance to show that low intravenous vitamin D intakes in the first week, shorter tibia length at 21 days and a long duration of TPN (≥16 days) are predictive for low BTT values at 36 weeks of GA.

Our study has some limitations. It is a prospective observational study so we can just observe and report our findings without drawing firm conclusions. Another aspect is that we did not provide data on the follow-up after hospital discharge of our newborns. Nonetheless, we think it is useful to analyze bone status in preterm IUGR and detect the postnatal nutritional management that best correlates with its improvement.

## 5. Conclusions

Our study suggests that IUGR infants could have a worse bone status at birth and a lower serum phosphate level in the first weeks of life despite adequate supplies.

Mc-BTT at 36 wGA, though not different between groups, correlated in IUGR preterms positively with basal phosphate, mean total energy of the first week and month, and negatively with days to reach full enteral feeding.

Low vitamin D intakes in the first week of life, shorter tibia length at 21 days and a long duration of parenteral nutrition (≥16 days) are predictive for lower BTT values at 36 weeks of GA.

Our study suggests that preterms, and in particular IUGR infants, need special nutritional care to promote bone development. A larger study, comprehensive of long term follow up, will clarify the possible influence of nutrition on long term bone health.

## Figures and Tables

**Table 1:** Clinical features and anthropometric parameters at birth.

	IUGR (*n* = 75) Mean ± s.d or Prevalence (Absolute Number)	Non-IUGR (*n* = 75) Mean ± s.d or Prevalence (Absolute Number)	*p* Level
Birth weight (g)	860 ± 260	1128 ± 255	**<0.001**
Length (cm)	34.3 ± 3.9	35.9 ± 2.9	**0.005**
Head circumference (cm)	25.1 ± 2.4	26.5 ± 2.0	**<0.001**
Lower limb length (mm)	79.80 ± 11.03	87.51 ± 11.47	**0.001**
Apgar at 5 min	7.7 ± 1.0	7.6 ± 1.0	0.554
Gestational age (days)	29.1 ± 2.1	28.9 ± 2.0	0.501
Sex (males)	47% (35)	41% (31)	0.204
Multiple pregnancy	26.6%	34.6%	0.376
Mode of delivery (CS)	97.3%	92.0%	0.276
Small for gestational age (SGA)	70.6% (53)	14.6% (11)	**<0.001**
Prenatal steroids			0.318
Complete course	86.6% (65)	78.6% (59)	
Incomplete course	8% (6)	16% (12)	
Mechanical ventilation + CPAP (days)	32.23 ± 33.86	20.17 ± 25.31	**0.020**
RDS	92.0% (69)	85.3% (64)	1.000
BPD 28 day	43% (32)	26% (19)	**0.039**
BPD 36 weeks GA	20% (15)	9% (6)	0.096
PDA	46.6% (35)	53.3% (40)	0.514
IVH	10.6% (8)	6.6% (5)	0.563
Sepsis	25.3% (19)	24.0% (18)	0.690
NEC	5.3% (4)	6.6% (5)	1.000
ROP	18.6% (14)	14.6% (11)	0.662
Cholestasis	18.6% (14)	13.3% (10)	0.480
EUGR (weight) at 36 weeks of GA	98.6% (73)	58.0% (43)	**<0.001**
Death	1.3% (1)	1.3% (1)	1.000
Days of Hospitalization	72.14 ± 36.41	57.30 ± 27.43	**0.009**

Abbreviations: CS: cesarean section; CPAP: continuous positive airway pressure; RDS: respiratory distress syndrome; BPD: bronchopulmonary dysplasia; PDA: patent ductus arteriosus; IVH: intraventricular hemorrhage; NEC: necrotizing enterocolitis; ROP: retinopathy of prematurity; GA: gestational age; EUGR: extrauterine growth restriction. Significant *p*-values are bold.

**Table 2:** Anthropometric, growth, biochemical and bone status parameters in the IUGR vs. non-IUGR groups.

	IUGR (*n* = 75) Mean ± s.d or Prevalence (Absolute Number)	Non-IUGR (*n* = 75) Mean ± s.d or Prevalence (Absolute Number)	*p* Level
Maximum weight loss (%)	9.74 ± 7.57	11.55 ± 6.39	0.133
Days to regain birth weight	9.91 ± 4.91	13.66 ± 5.74	**<0.001**
Minimum weight (g)	750.89 ± 211.19	965.62 ± 235.19	**<0.001**
Days to reach 1800 g	60.71 ± 21.77	45.02 ± 19.02	**<0.001**
Weight at 36 weeks GA (g)	1540 ± 298	1939 ± 309	**<0.001**
Head circumference at 36 weeks GA (cm)	30.24 ± 2.11	31.11 ± 2.59	0.084
Total length at 36 weeks GA (cm)	39.51 ± 2.99	42.69 ± 2.35	**<0.001**
Lower limb length at 36 weeks GA (mm)	98.1 ± 9.7	105.0 ± 8.3	**0.002**
Serum calcium at birth (mmol/L)	2.22 ± 0.24	2.05 ± 0.28	**<0.001**
Serum calcium at 21st day (mmol/L)	2.46 ± 0.13	2.44 ± 0.22	0.767
Serum phosphate at birth (mmol/L)	1.45 ± 0.44	1.79 ± 0.39	**<0.001**
Serum phosphate at 21st day (mmol/L)	1.76 ± 0.36	1.94 ± 0.25	**0.005**
Serum ALP at birth (IU/L)	169.69 ± 55.50	217.00 ± 77.89	**<0.001**
Serum ALP at 21st day (IU/L)	420.06 ± 146.31	370.54 ± 122.69	0.072
Basal mc-BTT (µs)	0.45 ± 0.10	0.51 ± 0.09	**<0.001**
mc-BTT at 21st day (µs)	0.43 ± 0.08	0.46 ± 0.09	**0.040**
mc-BTT at 36 weeks GA (µs)	0.48 ± 0.08	0.50 ± 0.07	0.160

Abbreviations: ALP: alkaline phosphatase; mc-BTT: metacarpal bone transmission time in µs; GA: gestational age. Significant *p*-values are bold.

**Table 3:** Nutritional parameters in the IUGR vs. non-IUGR groups.

	IUGR (*n* = 75) Mean ± s.d or Prevalence (Absolute Number)	Non-IUGR (*n* = 75) Mean ± s.d or Prevalence (Absolute Number)	*p* Level
Proteins intake 1st week iv (g/kg/day)	2.76 ± 0.51	2.68 ± 0.64	0.439
Proteins intake 2nd week iv (g/kg/day)	2.94 ± 0.73	2.74 ± 0.89	0.177
Mean total energy 1st week (kcal/kg/day)	73.42 ± 16.17	79.50 ± 16.25	**0.032**
Mean total energy 1st month	100.98 ± 14.88	106.37 ± 16.90	0.052
Total Parenteral Nutrition days	23.46 ± 13.10	16.04 ± 9.24	**<0.001**
Phosphorus intake 1st week iv (mmol/kg/day)	1.15 ±0.31	1.05 ± 0.28	**0.078**
Phosphorus intake 2nd week iv (mmol/kg/day)	1.47 ± 0.32	1.26 ± 0.39	**0.006**
Calcium intake in 1st week iv (mmol/kg/day)	0.83 ± 0.24	0.93 ± 0.28	**0.039**
Calcium intake in 2nd week iv (mmol/kg/day)	0.72 ± 0.31	0.72 ± 0.43	0.986
Vitamin D 1st week iv (IU/kg/day)	32.29 ± 14.58	45.09 ± 20.33	**<0.001**
Days to FEF (150 mL/kg/day)	27.13 ± 13.86	22.45 ± 13.34	**0.046**
Days of exclusive breast milk	28.74 ± 22.48	23.92 ± 21.37	0.332
Mother’s own milk at discharge	36% (26)	37% (25)	0.795

About nutritional parameters total energy intake regards both parenteral and enteral energy, expressed in kcal/kg/day; protein intake is expressed in g/kg/day; FEF: full enteral feeding. Significant *p*-values are bold.

**Table 4:** Correlations between anthropometric parameters and bone status.

		IUGR			Non-IUGR		
		mcBTT Basal	mcBTT 21 Day	mcBTT 36 Weeks GA	mcBTT Basal	mcBTT 21 Day	mcBTT 36 Weeks GA
Birth weight	r	0.597	0.494	0.331	0.711	0.502	0.462
	*p* value	**<0.001**	**<0.001**	**0.020**	**<0.001**	**<0.001**	**0.002**
Lower limb length at birth	r	0.593	0.336	0.192	0.248	0.030	0.481
	*p* value	**<0.001**	**0.024**	0.249	0.093	0.851	**0.006**
Weight 36 weeks GA	r	n.a	n.a.	0.541	n.a.	n.a.	0.204
	*p* value	n.a.	n.a.	**<0.001**	n.a.	n.a.	0.225
Lower limb length 36 weeks GA	r	n.a.	n.a.	0.428	n.a.	n.a.	0.086
	*p* value	n.a.	n.a.	**0.010**	n.a.	n.a.	0.645

Abbreviations: mc-BTT, metacarpal bone transmission time in µs; GA: gestational age; n.a.: not applicable. Significant *p*-values are bold.

**Table 5:** Correlations between nutritional and biochemical parameters and bone status in IUGR and non-IUGR preterms.

		IUGR		Non-IUGR	
		mcBTT 21 Day	mcBTT 36 wGA	mcBTT 21 Day	mcBTT 36wGA
Total mean energy intake 1st week (kcal/kg/day)	r *p* value	0.239 0.071	0.294 **0.040**	0.257 0.060	0.111 0.496
Total mean energy intake 1st month (kcal/kg/day)	r *p* value	0.470 **<0.001**	0.338 **0.017**	0.339 **0.011**	0.120 0.454
Vitamin D iv intake 1st week (UI/kg/day)	r *p* value	0.287 0.185	0.686 **<0.001**	0.042 0.849	0.484 **0.023**
TPN days	r *p* value	−0.428 **0.001**	−0.464 **0.001**	−0.296 **0.027**	−0.331 **0.032**
Days to reach FEF (150 mL/kg/day)	r *p* value	−0.436 **0.001**	−0.418 **0.004**	−0.221 0.104	−0.147 0.360
Basal serum phosphate (mmol/L)	r *p* value	0.283 **0.052**	0.341 **0.029**	0.200 0.163	0.079 0.643
Serum phosphate at 21st day (mmol/L)	r *p* value	0.182 0.249	0.359 **0.031**	0.453 **0.003**	0.484 **0.004**
Alkaline Phosphatase 21st day (IU/L)	r *p* value	−0.310 **0.043**	−0.045 0.790	−0.311 **0.033**	−0.300 **0.072**

Abbreviations: mcBTT: metacarpal bone transmission time in µs; wGA: weeks of gestational age; IU, International Unit; TPN: total parenteral nutrition; FEF: full enteral feeding. Significant *p*-values are bold.

**Table 6:** Lower limb length, vitamin D intake and energy cuts-off markers for bone status.

		mcBTT 36 Weeks GA		
	Cut-Off	Mean	sd	*p* Value
Lower limb length at 21st day (mm)	<91 ≥91	0.47 0.53	0.01 0.01	**<0.001**
Mean energy intake 1st week (Kcal/kg/day)	<77 ≥77	0.47 0.50	0.01 0.01	**0.039**
Mean energy intake 1st month (Kcal/kg/day)	<106 ≥106	0.47 0.51	0.01 0.01	**0.017**
VitD iv 1st week (IU/Kg/day)	<37 ≥37	0.45 0.52	0.01 0.01	**<0.001**
TPN days	<16 ≥16	0.53 0.46	0.01 0.01	**<0.001**

Abbreviations: mc-BTT, metacarpal bone transmission time in µs. IU, International Unit; TPN, total parenteral nutrition. Significant *p*-values are bold.

**Table 7:** Multivariate analysis of variance (MANOVA) for mc-BTT at 36 weeks of GA.

	Regression Coefficient	Standard Error	Prob. Level	Power (α = 0.05)
Intercept	0.4962	0.0221	/	/
Mean energy intake 1st week	0.0094	0.0205	0.5165	0.0983
TPN days	−0.0569	0.0194	**0.0041**	0.8377
VitD iv 1st week (IU/Kg/day)	0.0401	0.0200	**0.0268**	0.6090
Lower limb length at 21 days (mm)	0.0421	0.0186	**0.0302**	0.5901

Abbreviations: TPN: total parenteral nutrition; GA: gestational age. Significant Prob.Level are bold.

## Data Availability

The data presented in this study are available on request from the corresponding author.
